# Chloroplast genome structure in *Ilex* (Aquifoliaceae)

**DOI:** 10.1038/srep28559

**Published:** 2016-07-05

**Authors:** Xin Yao, Yun-Hong Tan, Ying-Ying Liu, Yu Song, Jun-Bo Yang, Richard T. Corlett

**Affiliations:** 1 Center for Integrative Conservation, Xishuangbanna Tropical Botanical Garden, Chinese Academy of Sciences, Mengla, 666303, China; 2 University of Chinese Academy of Sciences, Beijing, 100049, China; 3 Key Laboratory of Dai and Southern Medicine of Xishuangbanna Dai Autonomous Prefecture, Yunnan Branch of the Institute of Medicinal Plant Development, Chinese Academy of Medical Sciences, Jinghong, Yunnan, 666100, China; 4 Germplasm Bank of Wild Species, Kunming Institute of Botany, Chinese Academy of Sciences, Kunming, 650201, China

## Abstract

Aquifoliaceae is the largest family in the campanulid order Aquifoliales. It consists of a single genus, *Ilex*, the hollies, which is the largest woody dioecious genus in the angiosperms. Most species are in East Asia or South America. The taxonomy and evolutionary history remain unclear due to the lack of a robust species-level phylogeny. We produced the first complete chloroplast genomes in this family, including seven *Ilex* species, by Illumina sequencing of long-range PCR products and subsequent reference-guided *de novo* assembly. These genomes have a typical bicyclic structure with a conserved genome arrangement and moderate divergence. The total length is 157,741 bp and there is one large single-copy region (LSC) with 87,109 bp, one small single-copy with 18,436 bp, and a pair of inverted repeat regions (IR) with 52,196 bp. A total of 144 genes were identified, including 96 protein-coding genes, 40 tRNA and 8 rRNA. Thirty-four repetitive sequences were identified in *Ilex pubescens*, with lengths >14 bp and identity >90%, and 11 divergence hotspot regions that could be targeted for phylogenetic markers. This study will contribute to improved resolution of deep branches of the *Ilex* phylogeny and facilitate identification of *Ilex* species.

*Ilex*, in the monogeneric family Aquifoliaceae, is the largest woody dioecious genus in the angiosperms with approximately 600 species^1^. Flowers and fruits are fairly uniform but *Ilex* species differ greatly in leaf characters, including size, texture, and margins. The genus is widespread in mesic habitats but the global diversity centers are in East Asia and South America, with a single species in tropical Africa, two in northern Australia, four in Europe^1^, and 17 in North America (http://plants.usda.gov/java/nameSearch). The four other families in the Campanulidae order Aquifoliales, Cardiopteridaceae, Stemonuraceae, Helwingiaceae and Phyllonomaceae, are all small^2^,^3^. *Ilex* species are economically important sources of teas and medicines. *Ilex paraguariensis*, yerba mate, is planted on 326,000 ha in Argentina, Brazil and Paraguay, with a total annual production of more than a million tonnes^4^. In China, several species of *Ilex* are used to produce a popular medicinal tea, kuding cha^5^,^6^. *Ilex* species are also widely grown as ornamental plants because of their persistent red fruits and often distinctive leaves.

*Ilex* has a good fossil record dating to the Eocene and *Ilex*-like pollen has been reported from the Cretaceous, although molecular evidence suggests that the extant crown clade diverged only in the middle Miocene, 13 million years ago^1^,^7^. Phylogenetic relationships within *Ilex* are still unclear. Cuénoud^8^ used the chloroplast markers *atpB-rbcL* spacer and *rbcL* to construct a phylogeny of 116 species, while Manen^9^ combined plastid (*atpB-rbcL* spacer, *rbcL* and *trnL-trnF*) and nuclear (ribosomal internal transcribed spacer and the 5S RNA spacer) markers for 105 species. Manen^1^ later increased the phylogenetic resolution by using nuclear markers *ITS* and *ncpGS* for 108 species, including species from America, Europe, Africa, and islands in the Atlantic and Pacific. However, this study included only 33 species of the 204 known Chinese species, of which 149 are endemic^2^. These studies show a striking incongruence between plastid and nuclear phylogenies^1^. Similar incongruence has been reported in recent studies of the grass tribe Arundinarieae^10^, and the genera *Osmorhiza*^11^, *Hedyosmum*^12^, and *Medicago*^13^, and has been attributed to incomplete lineage sorting, plastid capture, or hybridization.

Chloroplasts contain a circular genome ranging from 21 kb in *Sciaphila densiflora*^14^ to 217 kb in *Pelargonium* × *hortorum*^15^, with two copies of a large inverted repeat (IR), one large single-copy region (LSC), and one small single-copy region (SSC)^16^,^17^. Despite the previous problems with using chloroplast sequences to construct a species-level phylogeny for *Ilex*, chloroplast sequences have advantages for species identification as a result of their small size, uniparental inheritance, haploid nature, and highly conserved genomic structure^16^. Species identification is a particular problem in *Ilex*, where there are many similar species. Moreover, since numerous copies are present in each cell, useable fragments of the chloroplast genome are more likely to persist in dried herbarium specimens^18^,^19^, which is an important consideration for *Ilex* in China where many species are only known from the type collection. In addition, chloroplast genomes have been used to improve the resolution of the backbone of phylogenies built with nuclear markers^10^.

Here, we present the complete chloroplast genomes of seven *Ilex* species through Illumina sequencing and reference-guided assembly of *de novo* contigs. We then test the feasibility of phylogeny reconstruction using chloroplast genomes in *Ilex*. Topologies of the phylogenies constructed from different molecular datasets are compared, including the whole cp genome, the coding regions, and LSC, SSC, IR, and introns and spacers. A new chloroplast genome for *Helwingia himalaica* (Yao *et al*., submitted), from the most closely related family, Helwingiaceae, is used as the outgroup.

## Results

### Output of genome sequencing and assembly

For the seven *Ilex* species, 239,377 to 748,662 paired-end reads (90bp in average reads length) were produced by Illumina sequencing. 233,558 to 692,191 reads were mapped to the reference genome *Camellia yunnanensis* (GenBank accession number KF156838), after screening these paired-end reads by aligning them to the reference genome, on average reaching over 100 × coverage of the cp genome. After *de novo* and reference-guided assembly, complete cp genomes of seven *Ilex* species were obtained. The four junction regions in each genome were validated using PCR-based sequencing (see Supplementary Table S1).

### Genome features and sequence divergence

**Figure 1:**
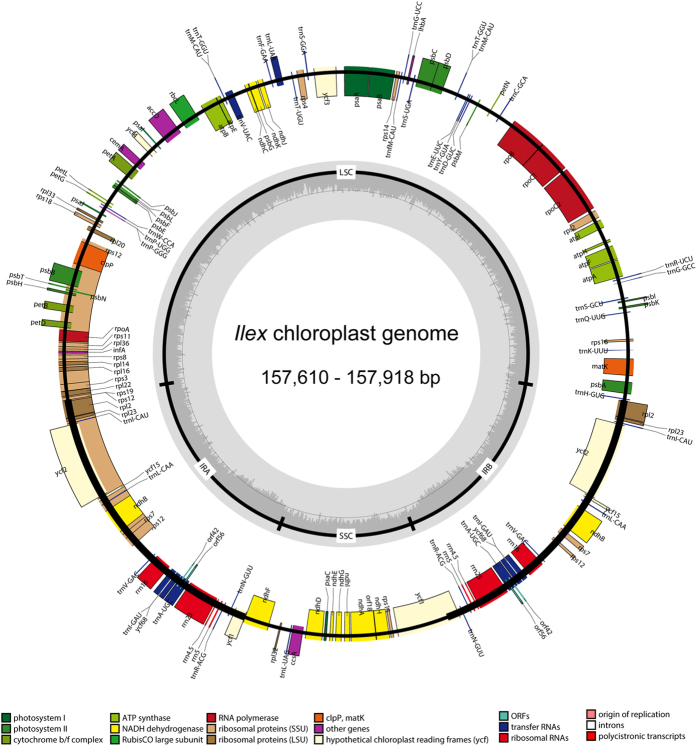
Gene map of the *Ilex* chloroplast genome. Genes shown outside the outer circle are transcribed clockwise and those inside are transcribed counterclockwise. Gray arrows indicate the direction of sequence coding. Genes belonging to different functional groups are color-coded. Dashed area in the inner circle indicates the GC content of the chloroplast genome.

Chloroplast genomes of the seven species were assembled into single circular, double-stranded DNA sequences, presenting a typical quadripartite structure including one large single-copy region (LSC with 86,948–87,266 bp), one small single-copy region (SSC with 18,427–18,513 bp), and a pair of inverted repeat regions (IR with 52,122–52,235 bp) (Table 1). The full length ranged from 157,610 in *I. latifolia* to 157,918 bp in *I. wilsonii* (Table 1). In *I. pubescens*, which was investigated in detail as an example, the chloroplast encoded a set of 144 genes of which 100 are unique genes and 16 are duplicated in the IRs regions (Fig. 1). The same gene order and clusters were found in all seven species. The 100 unique genes were composed of 74 protein-coding genes and 26 tRNA genes (Table 2). All of the eight rRNA genes were duplicated in the IR regions (Table 1). Nine distinct genes (*atp*F, *rpo*C1, *trn*L-UAA, *trn*V-UAC, *rpl*2, *ndh*B, *trn*I-GAU, *trn*A-UGC and *ndh*A) contain one intron and three genes (*ycf*3, *clp*P and *rps*12) have two introns. Gene *ycf*1 in the junction region between SSC and IRb was the only pseudogene found because of the incomplete duplication of the normal copy in the junction region (Fig. 1).

**Figure 2:**
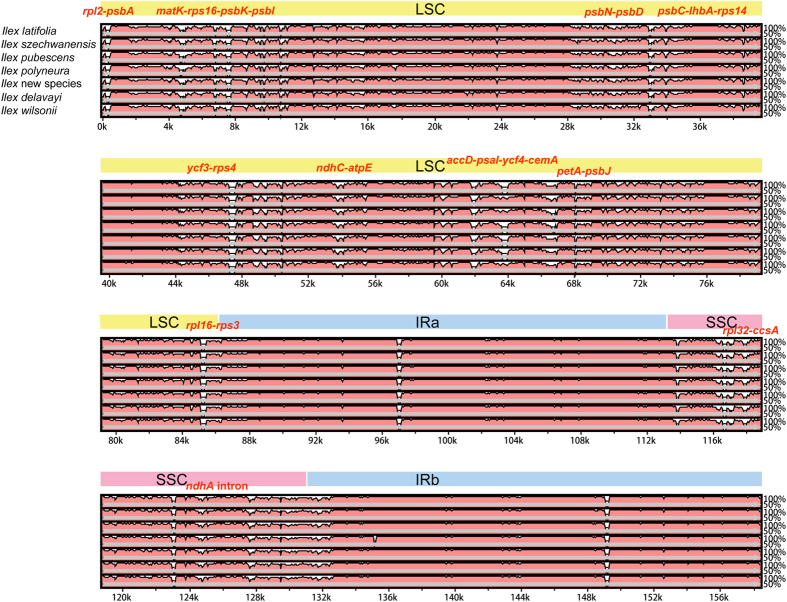
Visualization of the alignment of the seven *Ilex* chloroplast genomes. VISTA-based identity plots showing sequence identity with the *Helwingia himalaica* chloroplast genome as a reference. LSC indicates long single copy region; SSC indicates short single copy region; IRa and IRb indicate two inverted regions. Locations of divergent hotspot regions are labeled above alignment.

AT content is rich (62.3–62.4%) and sequence identity among the seven species was 97.9%. The whole aligned sequences disclosed moderate divergence with 11 regions containing sequence similarities below 50%, especially in intergenic regions. Eleven divergent hotspot regions were identified (Fig. 2). The *p*-distances between *Ilex* and *Helwingia,* and among *Ilex* species, were 0.03988 and 0.00288, respectively, both indicating moderate genetic divergences. The *p-*distance between the two most closely related species, *I. latifolia* and *I. delavayi* in subgenus *Ilex* section *Ilex*, was 0.00185.

### Indels and repeated sequences

A total of 113 indels were detected in the *Ilex* species, 88 in spacers, 13 in introns of genes, and 12 in genes, with 89 in LSC, 08 in SSC, and 08 in IRb (see Supplementary Table S1). In *I. pubescens* we identified 34 dispersed repeats >14 bp with sequence identify >90%, ranging from 15 bp to 29 bp (Table 3). Most repeats were 16 bp (32.4%) or 17 bp (23.5%). A total of 23 repeats were located in intergenic regions, while 06 were in protein-coding genes and 05 in tRNA genes. Five repeats were identified in *ycf2* which was the most in any gene.

### IRs region

Extensions of the IR into the genes *rps19* and *ycf1* were identified (Fig. 1): a small part of the 5′-end of *rps19* is in the IRb region and the 5′-end of *ycf1* extended into the IRa region, resulting in its pseudogenization due to the incomplete duplication.

### Genome divergence hotpot regions

Genome-wide comparative analyses among the seven *Ilex* species identified 11 hotspot regions for genome divergence that could be utilized as potential phylogenetic markers to reconstruct the phylogeny in this genus. These were *rpl2-psbA, matK-rps16-psbK-psbI, psbN-psbD, psbC-IhbA-rps14, ycf3-rps4, ndhC-atpE, accD-psaI-ycf4-cemA, petA-psbJ, rpl16-rps3, rpl32-ccsA*, and *ndhA* intron (Fig. 2). Character diversity of these hotspot regions was more than 4%.

### Phylogeny construction

**Figure 3:**
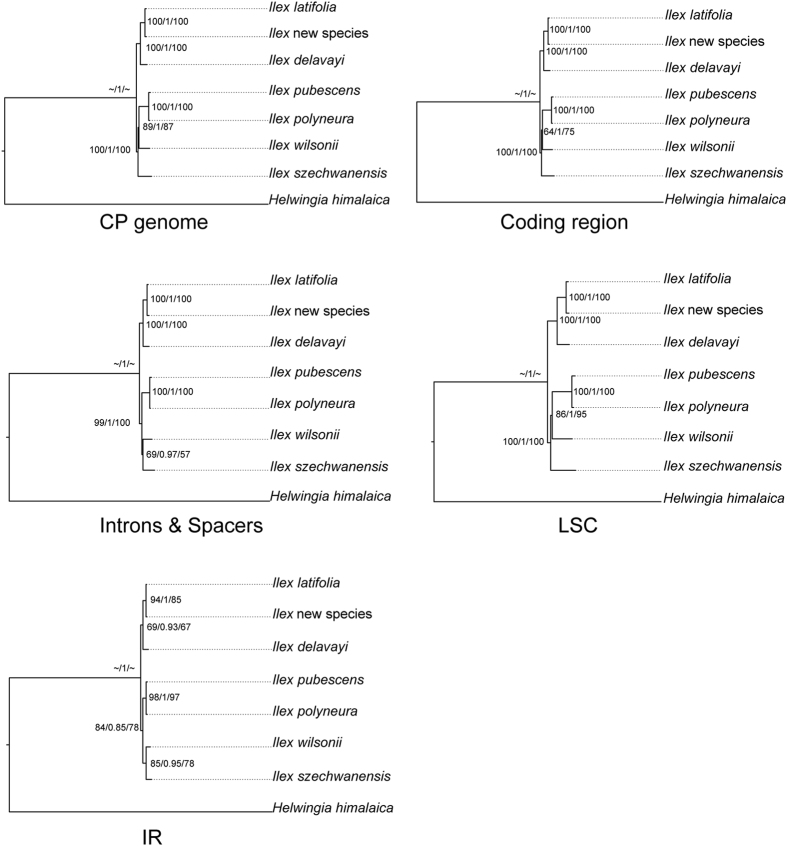
Phylogenetic relationships of the seven *Ilex* species and *Helwingia himalaica* constructed from chloroplast genes. Numbers near nodes indicate the maximum parsimony bootstrap (left) values for each clade present in the 50% majority-rule consensus tree, Bayesian posterior probability (middle), and maximum likelihood bootstrap (right) values for each clade present in the 50% majority-rule consensus tree.

**Figure 4:**
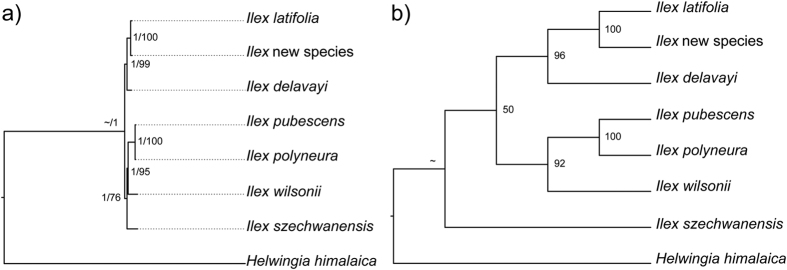
Phylogenetic relationships of the seven *Ilex* species and *Helwingia himalaica* based on the SSC region. In plot ‘a’ numbers near nodes indicate the Bayesian posterior probability (left) and maximum likelihood bootstrap (right) values for each clade present in the 50% majority-rule consensus tree; in plot ‘b’ numbers near nodes indicate the maximum parsimony bootstrap of each clade present in the 50% majority-rule consensus tree.

Among the cp genome sequences, protein-coding regions, LSC, SSC, IR, and introns and spacers, introns and spacers had the highest percentage variation at 1.7%, followed by LSC at 1.3%. The IR regions were least variable at 0.1%. The cp genome, SSC, and coding region, were 0.9%, 0.7% and 0.6%, respectively. Different methods of reconstructing phylogenies did not influence the topologic structure (Fig. 3) except with SSC, where maximum likelihood and Bayesian inference reconstructions differed in the position of *I. szechwanensis* from the one built by maximum parsimony (Fig. 4). However, in other respects all the phylogenies were the same, with *I. latifolia*, the new species, and *I. delavayi* forming one clade and the other species forming another. The cp genome and LSC phylogenies had higher bootstrap values and posterior probabilities than the others.

## Discussion

Modifications of chloroplast genome composition and gene order have been identified in many species in the asterid subclass Campanulidae, which includes the Aquifoliales, Asterales, Escalloniales, Bruniales, Apiales, Paracryphiales and Dipsacales. In this study, gene *ycf68* was found in *Ilex pubescens*, but not *Helwingia himalaica*. The cp genome of *Adenophora remotiflora* (Asterales) (KF889213 in GenBank) does not have *accD*, *clpP* and *infA*, while these genes are present in *Anethum graveolens* (Apiales)^20^, *Panax ginseng* (Apiales)^21^, *I. pubescens* and *H. himalaica* (Aquifoliales). *I. pubescens*, *H. himalaica* and *Panax ginseng* have lhbA, but *Adenophora remotiflora* and *Anethum graveolens* do not. In *Adenophora remotiflora* (KF889213 in GenBank) and *Trachelium caeruleum* (Asterales)^22^ genes *atpI*, *rps2*, *rpoC2*, *rpoC1* and *rpoB* are between *ycf3* and *rps12*, while in *Anethum graveolens* (Apiales)^20^, *Anthriscus cerefolium* (Apiales)^23^, *Tiedemannia filiformis* (Apiales)^23^, *Panax ginseng* (Apiales)^21^, *Schefflera delavayi* (Apiales)^24^, *Lonicera japonica* (Dipsacales) (NC_026839 in GenBank), and *I. pubescens* (Aquifoliales) they are between *atpH* and *trnC-GCA*. The distances between the locations of these five genes in the two groups are about 82,000 bp and their order is also different. Consequently, the total length of the cp genome differs between lineages in the Campanulidae.

In addition, variability in the extent of the inverted repeat (IR) regions has been found, with the boundaries between IR and LSC or SSC very fluid. Gene *rps19* is nearest to the LSC-IR boundary: in some species, like *I. pubescens*, *H. himalaica*, and *Panax ginseng*^21^, it spans the boundary, in others, like *Millettia pinnata*^25^ and *Lupinus luteus*^26^, it does not extend into the IR, while in others, like *Phaseolus vulgaris*^27^, *Vigna radiata*^28^ and *Vigna unguiculata* (JQ755301 in GenBank), the whole gene is inside the IR. Gene *ycf1*, nearest to the SSC-IR boundary, is similar.

*I. pubescens* had fewer and smaller dispersed repeats than reported for some other campanulids. The largest was 29 bp, while in the Apiales 9–29 repeats >30 bp were recorded in various species, with the largest 79 bp in length^23^.

Variable plastid regions have been used to design markers to investigate phylogenetic relationships, for instance *rbcL*, *matK*, and *atpB*, which have been widely used in phylogenic reconstruction from the genus level upwards. However, these genes are most divergent and informative among distantly related species, and are not suited for studying relationships between species in the same genus, like *Curcuma*^29^ and some genera in the Lauraceae^30^. According to the alignment of the cp genome of seven *Ilex* species studied here, *rbcL*, *matK*, and *atpB* were not appropriate for studies within *Ilex* because their divergences, which were 0.2%, 0.16% and 0.07%, respectively, were too low. However, 11 divergent regions were identified with sequence divergences around 4%.

Divergent hotspot regions in the chloroplasts are particularly useful for species-level identification in *Ilex*, which has many similar species represented by few collections. For example, several Chinese species, including *Ilex chengkouensis* C. J. Tseng, *Ilex euryoides* C. J. Tseng, *Ilex synpyrena* C. J. Tseng, and *Ilex ningdeensis* C. J. Tseng, have only been collected once. Moreover, some species currently recognized, such as *Ilex huoshanensis* Y. H. He, *Ilex dabieshanensis* K. Yao & M. P. Deng, *Ilex urceolatus* C. B. Shang, K. S. Tang & D. Q. Du, and *Ilex wugonshanensis* C. J. Tseng ex S. K. Chen & Y. X. Feng, are not clearly distinct in morphology and distribution from their nearest relatives, and may not deserve species status. A study of randomly sampled herbarium specimens in the National Herbarium in Beijing found that, although the DNA was usually highly degraded and most fragment <300 bp, it was still possible to extract usable genetic material from around a third of specimens^18^. This suggests that similar techniques could be used to clarify the diversity and status of the rare and little-known *Ilex* species in China.

For the phylogenetic reconstructions presented here, most informative characters occurred in intergenic regions, with some of these identified as divergent hotspot regions, as was also shown in *Camellia*^16^ and the Bambusoideae^31^. Among the phylogenies built by six different subsets of the genomic data, trees based on the complete cp genome and LSC displayed the highest support, although most nodes had high support in all. The topologies of different phylogenies were very similar, as also shown in *Camellia* and the Bambusoideae. These studies also showed that the methods used to build the phylogeny (MP, ML or BI) had a relatively minor influence.

The results of our phylogenetic analyses agree in part with the traditional classification system used in the Flora of China^2^. *I. delavayi* and *I. latifolia* in section *Aquifolium* form one clade with a new, large-fruited species, which is similar to but distinct from *I. latifolia* (Tan *et al*., submitted). However, in the other clade, the evergreen species *I. pubescens* and *I. wilsonii* are in section *Pseudoaquifolium*, while the deciduous *I. polyneura* is in *Micrococca*. In the classification used in the FOC all the deciduous species form a separate clade. Data from nuclear genes will be needed to resolve these differences, as shown previously for *Ilex* by Manen^1^. The chloroplast genome is also expected to be useful in helping to resolve the deeper branches of the phylogeny as more whole-genome sequences become available.

## Conclusions

The chloroplast genomes of seven *Ilex* species are reported for the first time in this study and their organization is described and compared with that of other campanulids. Eleven divergent regions were identified, which can be used to develop phylogenetic markers. Phylogenies were constructed using the entire genomes and various subsets and their topologies and resolutions compared. Our results will be useful for identification at the species level and for helping to resolve the deeper branches of the phylogeny.

## Methods

### Plant materials

Plant materials used in this study were intact, fresh, young leaves collected in Yunnan. Species were identified with the Flora of China^2^ and specimens were deposited in the herbarium of Xishuangbanna Tropical Botanical Garden, Chinese Academy of Sciences (HITBC) (Table 4).

### Chloroplast genome sequencing and assembly

About 100 mg of fresh leaf material of each species was used to extract total DNA by a modified CTAB method^17^,^32^, with 4% CTAB with 0.2% DL-dithiothreitol (DTT) replacing 2% CTAB, and adding approximately 1% polyvinyl polypyrrolidone (PVP) while milling the materials. Long-range PCR was used for DNA amplification of the plastome using nine universal primers developed by Yang^17^. Each amplification was performed in 25 μL of a reaction mixture containing 1 × PrimeSTAR GXL buffer (10 mM Tris-HCl (pH 8.2), 1 mM MgCl_2_, 20 mM NaCl, 0.02 mM EDTA, 0.02 mM DTT; 0.02% Tween 20, 0.02% Nonidet P-40, and 10% glycerol); 1.6 mM of dNTPs, 0.5 μM of each primer; 1.25 U of Prime-STAR GXL DNA polymerase (TAKARA BIO INC., Dalian, China), and 30–100 ng of DNA template. The amplification was conducted using 94 °C for 1 min, 30 cycles of 98 °C for 10 s and 68 °C for 15 min, followed by a final extension step at 72 °C for 10 min.

The 6 μg PCR product was fragmented for constructing short-insert (500 bp) libraries according to the Illumina manual, using the Illumina Nextera XT library (Illumina, San Diego, CA, USA). DNA from each individual was indexed using tags and pooled together in one lane of a Illumina Hiseq 2000 for sequencing at the Germplasm Bank of Wild Species in Southwest China, Kunming Institution of Botany, Chinese Academy of Sciences.

Raw reads were filtered by quality control software NGSQCToolkit v2.3.3^33^ to obtain high quality Illumina data (cut-off value for percentage of read length = 80, cut-off value for PHRED quality score = 30) and vector- and adaptor-free reads. Filtered reads were then assembled into contigs in the software CLC Genomics Workbench 8 (http://www.clcbio.com), by *de novo* method using a *k*-mer of 63 and a minimum contig length of 1 kb. Outputted contigs were aligned with a reference *Camellia yunnanensis* chloroplast genome (Genbank accession number KF156838), which was the most similar genome identified via BLAST (http://blast.ncbi.nlm.nih.gov/), and ordered according to the reference genome. Contigs were aligned with the reference genome for assembly of the chloroplast genome of each species in Geneious 4.8^34^. Lastly, junctions between LSC/IRs and SSC/IRs were validated by Sanger sequencing of PCR-based products using newly designed primers (see Supplementary Table S2).

### Genome annotation and repeat analysis

Assembled genomes were annotated using the Dual Organellar GenoMe Annotator (DOGMA) database^35^, then manually edited for start and stop codons. All annotated cp genomes will be deposited in GenBank. Genome maps were drawn in OGDraw 1.2^36^. Multiple sequence alignment was done with MAFFT 5^37^ and manually edited where necessary. A comparative plot of full alignment with annotation of these eight genomes was produced by mVISTA^38^,^39^, using *Helwingia himalaica* as a reference. REPuter was used to detect and assess repeats^40^ in *I. pubescens*. Average genetic divergences of these eight species were estimated using *p-*distances. The genetic divergence between the two most closely related species, *I. latifolia* and *I. delavayi*, was also estimated.

### Molecular marker identification

In order to explore the divergence of chloroplast genes in *Ilex* and its utilization in identification, all coding genes, introns and spacers were extracted. Every homologous region was aligned by MUSCLE^41^ and manually edited where necessary.

### Phylogenetic analyses

Sequences of the seven *Ilex* species and *Helwingia himalaica* were aligned using MAFFT^37^ and manually edited where necessary. Unambiguously aligned DNA sequences were used for phylogeny construction. Phylogenies were constructed by maximum parsimony (MP), maximum likelihood (ML) and Bayesian Inference analyses (BI) using the entire cp genome and also using exons of protein-coding regions, introns and spacers, LSC, SSC, and IR. Lengths of all alignment matrices of these datasets are shown in Supplementary Table S3. In all phylogenetic analyses, *Helwingia himalaica* was used as outgroup.

MP and ML analyses were conducted in PAUP 4.0b10^42^. For MP analysis, heuristic searches were conducted with tree bisection-reconnection (TBR) branch swapping, with the ‘Multrees’ option in effect. Bootstrap analysis was conducted with 1,000 replicates with TBR branch swapping. For ML analysis, the best substitution model was tested according to the Akaike information criterion (AIC) by jModeltest version 2^43^,^44^ (see Supplementary Table S3). BI analysis was conducted using MrBayes version 3.2.2^45^ and the best substitution model tested by AIC. Two independent Markov Chain Monte Carlo chains were calculated simultaneously for 10,000,000 generations and sampled every 1,000 generations. Potential Scale Reduction Factor (PSRF) values were used to determine convergence in Bayesian Inference using MrBayes version 3.2.2^45^. All PSRF values were 1, indicating that these analyses converged. The first 25% of calculated trees was discarded as burn-in and a consensus tree constructed using the remaining trees.

## Additional Information

**How to cite this article**: Yao, X. *et al*. Chloroplast genome structure in *Ilex* (Aquifoliaceae). *Sci. Rep.* **6**, 28559; doi: 10.1038/srep28559 (2016).

## Supplementary Material

Supplementary Tables

## Figures and Tables

**Table 1:** Comparison of plastid genomic characteristics in seven *Ilex* species and *Helwingia himalaica*.

	*Ilex latifolia*	*Ilex szechwanensis*	*Ilex pubescens*	*Ilex polyneura*	*Ilex*new sp.	*Ilex delavayi*	*Ilex wilsonii*	*Helwingia himalaica*
Total paired-end reads	1,057,844	1,292,586	1,135,472	467,116	1,338,864	1,201,978	1,459,906	914,296
Aligned paired-end reads	1,045,069	1,257,399	1,131,722	465,040	1,327,085	1,198,537	1,448,390	907,908
Mean coverage	143.8	523.2	504	523.2	1006.0	562.3	342.6	188.2
Number of contigs	230	84	68	47	44	76	106	142
Mean length (bp)	1,649	2,419	2,834	3,505	3,654	2,564	1,982	1,820
N50 (bp)	2,806	8,762	9,089	9,503	9,506	8,343	8,346	4,534
Sum contigs length (bp)	379,388	203,271	192,740	164,767	160,803	194,865	210,138	258,537
Size (bp)	157,610	157,900	157,741	157,621	157,611	157,671	157,918	158,362
LSC length (bp)	86,952	87,204	87,109	87,064	86,948	87,000	87,266	87,810
SSC length (bp)	18,429	18,513	18,436	18,435	18,434	18,436	18,432	18,560
IRs length (bp)	52,229	52,183	52,196	52,122	52,229	52,235	52,220	51,992
Protein Genes [unique]	96[74]	96[74]	96[74]	96[74]	96[74]	96[74]	96[74]	94[76]
tRNA [unique]	40[26]	40[26]	40[26]	40[26]	40[26]	40[26]	40[26]	40[26]
rRNA [unique]	8[0]	8[0]	8[0]	8[0]	8[0]	8[0]	8[0]	8[0]
GC content (%)	37.60	37.70	37.60	37.60	37.60	37.60	37.60	37.70

**Table 2:** List of genes in the chloroplast genome of *Ilex.*

Category	Groups of gene	Name of genes
Protein synthesis and DNA-replication	Transfer RNAs	*trnC-GCA, trnD-GUC, trnE-UUC, trnF-GAA, trnfM-CAU, trnG-GCC, trnG-UCC, trnH-GUG, trnK-UUU, trnL-UAA, trnM-CAU, trnQ-UUG, trnP-GGG, trnP-UGG, trnR-UCU, trnS-GCU, trnS-GGA, trnS-UGA, trnT-GGU, trnT-UGU, trnV-UAC, trnW-CCA, trnY-GUA, trnA-UGC*(×2)*, trnI-CAU*(×2)*, trnI-GAU*(×2)*, trnL-CAA*(×2)*, trnL-UAG, trnN-GUU*(×2)*, trnR-ACG*(×2)*, trnV-GAC*(×2)
	Ribosomal RNAs	*rrn16*(×2)*, rrn23*(×2)*, rrn4.5*(×2)*, rrn5*(×2)
	Ribosomal protein small subunit	*rps16, rps2, rps14, rps4, rps18, rps12*(×2)*, rps11, rps8, rps3, rps19, rps7*(×2)*, rps15*
	Ribosomal protein large subunit	*rpl33, rpl20, rpl36, rpl14, rpl16, rpl22, rpl2*(×2)*, rpl23*(×2)*, rpl32*
	Subunits of RNA polymerase	*rpoA, rpoB, rpoC1, rpoC2*
Photosynthesis	photosystem I	*psaA, psaB, psaC, psaI, psaJ*
	Photosystem II	*psbA, psbB, psbC, psbD, psbE, psbF, psbG, psbH, psbI, psbJ, psbK, psbL, psbM, psbN, psbT, lhbA*
	Cythochrome b/f complex	*petA, petB, petD, petG, petL petN*
	ATP synthase	*atpA, atpB, atpE, atpF, atpH, atpI*
	NADH-dehydrogenase	*ndhA, ndhB*(×2)*, ndhC, ndhD, ndhE, ndhF, ndhG, ndhH, ndhI, ndhJ, ndhK*
	Large subunit Rubisco	*rbcL*
Miscellaneous group	Translation initiation factor	*infA*
	Acetyl-CoA carboxylase	*accD*
	Cytochrome c biogenesis	*ccsA*
	Maturase	*matK*
	ATP-dependent protease	*clpP*
	Inner membrane protein	*cemA*
Pseudogene unknown function	Conserved hypothetical chloroplast ORF	*ycf3, ycf4, ycf2*(×2)*, ycf15*(×2)*, ycf68*(×2)*, orf42*(×2)*, orf56*(×2)*, ycf1*(×2)*, orf188*

**Table 3:** Repetitive sequences of *Ilex pubescens* calculated in REPuter.

Repeat length (bp)	Repeat bases	Repeat location	Copy of repeat location
15	TCTTTCTTTTTTTTT	*trnH-GUG psbA spacer*	*clpP*
16	TTTTTTTTTTTTTTTT	*trnH-GUG psbA spacer*	*trnM-CAU atpE spacer*
16	TTGAAAAAAAAAAAAA	*atpA-atpF spacer*	*trnS-UGA lhbA spacer*
16	TGAAAAAAAAAAAAAA	*atpA-atpF spacer*	*rps18-rpl20 spacer*
16	ATTTCTTTTTTAGTTT	*atpH-atpI spacer*	*ycf1*
16	TTTTTTGAAAAAAAAA	*rps2-rpoC2 spacer*	*ndhF-rpl32 spacer*
16	GAAAAAAAAAAAAAGA	*lhbA trnG-UCC spacer*	*rps18-rpl20 spacer*
16	AAAAAAAAAAAAAGAA	*lhbA trnG-UCC spacer*	*rpl14-rpl16 spacer*
16	ATTATTAATTTGTATG	*trnF-GAA ndhJ spacer*	*ycf1*
16	TAGTCACTTCTTTTTT	*psaJ-rpl33 spacer*	*ycf2*
16	CTTTCTTTTTTTTTTC	*clpP*	*infA-rps8 spacer*
16	ATTTTTATTTTGTTTT	*rpl16-rps3 spacer*	*rps19-rpl2 spacer*
17	TTTTTTTTTTTTTTATT	*trnH-GUG psbA spacer*	*psbE-petL spacer*
17	CTTTTTTGAAAAAAAAA	*atpA-atpF spacer*	*rps2-rpoC2*
17	TTTTTTGAAAAAAAAAA	*atpA-atpF spacer*	*ndhF-rpl32*
17	TAGTAAAAATAAAAAGA	*trnM-CAU psbD spacer*	*accD-psaI spacer*
17	AAGACGAAAAAAAAAAA	*trnT-UGU trnL-UAA spacer*	*petA-psbJ spacer*
17	CTATATATTTTTCCAGT	*cemA-petA spacer*	*petD*
17	GCTTTTGTTTATAAAAA	*rpl16-rps3 spacer*	*rpl16-rps3 spacer*
17	GATATTGATGCTAGTGA	*ycf2*	*ycf2*
18	TCCACTCAGCCATCTCTC	*trnS-GCU*	*trnS-UGA*
18	CGAAAATTCTTTTTTCTC	*trnE-UUC trnM-CAU spacer*	*rpl32 trnL-UAG spacer*
18	ATTGTATCCATTGAGCAA	*psaB*	*psaA*
18	ATGCAATAGCTAAATGAT	*psaB*	*psaA*
18	CTTTTCTGAGTGAACTAG	*accD*	*accD*
18	AGAACTACGAGATCACCC	*trnI-GAU*	*trnA-UGC*
19	TGCGGGTTCGATTCCCGCT	*trnG-GCC*	*trnG-UCC*
21	ATGCTGCTGCAGAATAAACCA	*trnH-GUG psbA spacer*	*rpl22*
21	AAGAGAGGGATTCGAACCCTC	*trnS-GCU*	*trnS-UGA*
21	AGACAGGATTTGAACCCGTGA	*trnfM-CAU*	*trnP-UGG*
23	TCATTGTTCCACTCTTTGACAAC	*rrn4.5-rrn5 spacer*	*rrn4.5-rrn5 spacer*
26	GTGAGATTTTCATCTCATACGGCTCC	*ycf3*	*ndhA*
26	TTATTTATTTTATATTCTATTTCAAT	*rps4 trnT-UGU spacer*	*rps4 trnT-UGU spacer*
29	TCGATATTGATGATAGTGACGATATTGAT	*ycf2*	*ycf2*

**Table 4:** Sampled species and their voucher specimens used in this study according to the taxonomic treatment in the Flora of China.

Species	Subgenus	Section	Geographic origin	Voucher	Accession number in GenBank
*Ilex latifolia*	*Ilex*	*Aquifolium*	XTBG	YX1303	KX426465
*Ilex szechwanensis*	*Ilex*	*Paltoria*	Dali, Yunnan	YX1418	KX426466
*Ilex pubescens*	*Ilex*	*Pseudoaquifolium*	Xishuangbanna, Yunnan	YX1676	KX426467
*Ilex polyneura*	*Prinos*	*Micrococca*	Xishuangbanna, Yunnan	YX1680	KX426468
*Ilex* new species	–	–	Xishuangbanna, Yunnan	YX1681	KX426469
*Ilex delavayi*	*Ilex*	*Aquifolium*	Dali, Yunnan	YX1723	KX426470
*Ilex wilsonii*	*Ilex*	*Pseudoaquifolium*	Yichun, Jiangxi	YX1748	KX426471
*Helwingia himalaica*	–	–	Gongshan, Nujiang	YX1678	KX434807
