# Supplementary material for: Chloroplast genome structure in Ilex (Aquifoliaceae)
Source: Sci Rep. 2016 Jul 5;6:28559. doi: 10.1038/srep28559 (PMC4932625; doi:10.1038/srep28559)
Supplement: Supplementary Tables [file srep28559-s1.pdf]

Chloroplast genome structure in *Ilex* (Aquifoliaceae)

Xin Yao[1,2+], Yun-hong Tan[1+], Ying-ying Liu[3], Yu Song[1,2], Jun-bo Yang[4*], and Richard T. Corlett[1*]

[1]Center for Integrative Conservation, Xishuangbanna Tropical Botanical Garden, Chinese Academy of Sciences, Mengla, 666303, China

[2]University of Chinese Academy of Sciences, Beijing, 100049, China

[3]Key Laboratory of Dai and Southern Medicine of Xishuangbanna Dai Autonomous Prefecture, Yunnan Branch of the Institute of Medicinal Plant Development, Chinese Academy of Medical Sciences, Jinghong, Yunnan, 666100, China

[4]Germplasm Bank of Wild Species, Kunming Institute of Botany, Chinese Academy of Sciences, Kunming, 650201, China

*Correspondence: jbyang@mail.kib.ac.cn; corlett@xtbg.org.cn

[+]These authors contributed equally to this work

No.	Star locus	*Ilex latifolia*	*Ilex* new sp.	*Ilex delavayi*	*Ilex szechwanensis*	*Ilex pubescens*	*Ilex polyneura*	*Ilex wilsonii*	Location	Region
1	167	d[§]	d	d	d	d	d	i	*trnH-GUG(psbA)*	LSC
2	242	d	d	d	i	i	i	i	*trnH-GUG(psbA)*	LSC
3	359	i	i	i	d	i	i	i	*trnH-GUG(psbA)*	LSC
4	424	i	i	i	d	i	i	i	*trnH-GUG(psbA)*	LSC
5	579	i	i	i	i	i	i	d	*trnH-GUG(psbA)*	LSC
6	1708	i	i	d	i	i	i	i	*trnH-GUG(psbA)*	LSC
7	1865	i	i	i	d	i	i	i	*psbA(matK)*	LSC
8	3907	d	d	d	d	d	i	d	*matK(trnK-UUU)*	LSC
9	4749	i	i	i	i	d	d	d	*trnK-UUU(rps16)*	LSC
10	4936	i	i	i	d	i	i	i	*trnK-UUU(rps16)*	LSC
11	4980	i	i	i	i	d	i	i	*trnK-UUU(rps16)*	LSC
12	4998	i	i	i	d	i	i	i	*trnK-UUU(rps16)*	LSC
13	5310	i	i	i	i	i	i	d	*trnK-UUU(rps16)*	LSC
14	5855	d	i	i	i	i	i	i	*rps16(trnQ-UUG)*	LSC
15	5912	i	i	i	d	d	d	i	*rps16(trnQ-UUG)*	LSC
16	5957	i	i	i	i	d	d	i	*rps16(trnQ-UUG)*	LSC
17	6643	i	i	i	d	i	i	i	*rps16(trnQ-UUG)*	LSC
18	7452	i	i	i	d	i	i	i	*rps16(trnQ-UUG)*	LSC
19	8603	i	i	i	d	i	i	i	*psbK*	LSC
20	8754	d	d	i	d	d	d	d	*psbK*	LSC
21	8946	i	i	i	i	i	i	d	*psbK(psbI)*	LSC
22	9058	i	i	d	i	i	i	i	*psbK(psbI)*	LSC
23	9443	i	i	i	d	i	i	i	*psbI(trnS-GCU)*	LSC
24	9671	d	d	d	i	d	d	i	*trnS-GCU(trnG-GCC)*	LSC
25	11050	d	d	i	d	d	d	d	*trnS-GCU(trnG-GCC)*	LSC
26	11180	i	i	d	i	i	i	i	*trnR-UCU(atpA)*	LSC
27	11197	d	d	d	i	d	d	i	*trnR-UCU(atpA)*	LSC
28	13816	i	i	i	d	i	i	i	*atpF intron*	LSC
29	15167	i	i	d	d	d	d	d	*atpH(atpI)*	LSC
30	15192	d	d	d	d	d	d	i	*atpH(atpI)*	LSC
31	17760	i	i	i	i	i	d	i	*rps2(rpoC2)*	LSC
32	22125	i	i	d	i	i	i	i	*rpoC2*	LSC
33	28661	i	i	i	d	i	i	i	*rpoB(trnC-GCA)*	LSC
34	30267	i	i	i	d	i	i	i	*trnC-GCA(petN)*	LSC
35	30778	d	d	i	i	i	i	i	*petN(psbM)*	LSC
36	30822	d	d	i	d	d	d	d	*petN(psbM)*	LSC
37	31057	i	i	i	d	i	i	i	*petN(psbM)*	LSC
38	32362	i	i	d	d	i	i	d	*psbM(trnD-GUC)*	LSC
39	32477	d	d	d	d	d	d	i	*trnD-GUC*	LSC
40	33060	d	d	d	i	d	d	d	*trnE-UUC(trnT-GGU)*	LSC
41	33072	d	d	i	d	d	d	d	*trnE-UUC(trnT-GGU)*	LSC

42	33120	d	d	d	d	i	i	i	*trnE-UUC(trnT-GGU)*	LSC
43	33235	i	i	d	i	i	i	i	*trnE-UUC(trnT-GGU)*	LSC
44	33507	d	d	d	i	i	i	i	*trnE-UUC(trnT-GGU)*	LSC
45	34449	i	i	d	i	i	i	i	*trnT-GGU(psbD)*	LSC
46	38038	i	i	i	i	d	d	i	*trnS-UGA(lhbA)*	LSC
47	44226	i	i	i	i	d	i	i	*psaA(ycf3)*	LSC
48	46143	d	d	d	d	d	d	i	*ycf3* intron2	LSC
49	46663	i	i	i	i	i	d	d	*ycf3* intron2	LSC
50	46666	i	i	d	i	d	d	d	*ycf3* intron2	LSC
51	47067	d	d	d	i	d	d	d	*ycf3(trnS-GGA)*	LSC
52	47240	i	i	i	d	i	i	i	*ycf3(trnS-GGA)*	LSC
53	48367	d	d	d	i	i	i	d	*rps4(trnT-UGU)*	LSC
54	48460	d	d	d	d	d	d	i	*rps4(trnT-UGU)*	LSC
55	48559	i	i	i	d	i	i	i	*rps4(trnT-UGU)*	LSC
56	48935	i	i	i	i	i	i	d	*trnT-UGU(trnL-UAA)*	LSC
57	49052	i	i	i	d	i	i	i	*trnT-UGU(trnL-UAA)*	LSC
58	49137	i	i	d	i	i	i	i	*trnT-UGU(trnL-UAA)*	LSC
59	49212	i	i	i	d	d	d	d	*trnT-UGU(trnL-UAA)*	LSC
60	49865	d	d	d	d	i	i	d	*trnL-UAA* intron	LSC
61	49965	d	d	d	d	d	d	i	*trnL-UAA* intron	LSC
62	50078	i	i	d	i	i	i	i	*trnL-UAA* intron	LSC
63	50429	d	d	i	i	i	i	i	*trnL-UAA(trnF-GAA)*	LSC
64	51315	d	d	d	d	i	i	d	*trnf-GAA(ndhJ)*	LSC
65	53169	i	i	d	i	i	i	i	*ndhC(trnV-UAC)*	LSC
66	53294	d	d	d	i	d	d	i	*ndhC(trnV-UAC)*	LSC
67	53373	i	i	i	i	i	i	d	*ndhC(trnV-UAC)*	LSC
68	59647	i	i	i	i	d	d	i	*rbcL(accD)*	LSC
69	59804	i	i	i	d	i	i	i	*rbcL(accD)*	LSC
70	59905	i	i	i	i	d	i	i	*rbcL(accD)*	LSC
71	60485	i	i	i	d	i	i	i	*accD*	LSC
72	62033	d	d	d	d	d	d	i	*accD(psaI)*	LSC
73	62655	i	i	i	d	i	i	d	*psaI(ycf4)*	LSC
74	63619	i	i	d	i	i	i	i	*ycf4(cemA)*	LSC
75	63905	d	d	d	i	i	i	i	*ycf4(cemA)*	LSC
76	66908	i	i	i	d	i	i	i	*petA(psbJ)*	LSC
77	66971	d	i	i	i	i	i	i	*petA(psbJ)*	LSC
78	66975	i	i	i	i	d	d	i	*petA(psbJ)*	LSC
79	69461	i	d	i	i	i	i	i	*petL(petG)*	LSC
80	69788	d	d	d	i	d	d	d	*petG(trnW-CCA)*	LSC
81	73500	i	i	i	d	d	d	d	*rps12* intron1	LSC
82	75167	i	i	i	d	i	i	i	*clpP* intron2	LSC
83	78864	d	d	i	d	d	d	d	*psbH(petB)*	LSC
84	80061	i	i	i	i	i	i	d	*petB(petD)*	LSC
85	80811	d	i	i	i	i	i	i	*petB(petD)*	LSC

86	83236	i	i	d	d	d	d	d	*rps11(rpl36)*	LSC
87	84398	i	i	i	i	i	d	i	*rpl36*	LSC
88	85488	i	d	i	d	i	i	i	*rpl16(rps3)*	LSC
89	86339	d	d	d	i	i	i	i	*rpl16(rps3)*	LSC
90	97804	i	i	i	d	i	i	i	*trnL-CAA(ndhB)*	IRb
91	97912	i	i	i	d	i	i	i	*trnL-CAA(ndhB)*	IRb
92	98080	i	i	i	i	d	d	i	*trnL-CAA(ndhB)*	IRb
93	103072	i	i	i	d	i	i	i	*rps12* intron2	IRb
94	103125	d	d	d	d	i	d	d	*rps12* intron2	IRb
95	111186	i	i	i	i	i	d	i	*rrn4.5*	IRb
96	112381	i	i	i	d	i	i	i	*trnR-ACG(trnN-GUU)*	IRb
97	113834	i	i	i	i	i	i	d	*ycf1*	IRb
98	116932	i	i	i	d	i	i	i	*ndhF(rpl32)*	SSC
99	117489	d	i	i	i	i	i	i	*rpl33(trnL-UAG)*	SSC
100	117563	d	i	i	i	i	i	i	*rpl34(trnL-UAG)*	SSC
101	118224	d	d	d	i	d	d	d	*trnL-UAG*	SSC
102	124467	d	d	d	d	d	d	i	*ndhA* intron	SSC
103	124669	d	d	d	d	d	d	i	*ndhA* intron	SSC
104	127363	i	i	i	i	i	i	d	*rps15*	SSC
105	127403	i	i	i	d	i	i	i	*rps15*	SSC
106	132661	i	i	i	i	i	i	d	*ycf1*	IRa
107	134111	i	i	i	d	i	i	i	*trnN-GUU(trnR-ACG)*	IRa
108	135248	i	i	i	i	i	d	i	*rrn5(rrn4.5)*	IRa
109	143358	d	d	d	d	i	d	d	*trnV-GAC(rps7)*	IRa
110	143420	i	i	i	d	i	i	i	*trnV-GAC(rps7)*	IRa
111	148415	i	i	i	i	d	d	i	*ndhB(trnL-CAA)*	IRa
112	148584	i	i	i	d	i	i	i	*ndhB(trnL-CAA)*	IRa
113	148683	i	i	i	d	i	i	i	*ndhB(trnL-CAA)*	IRa

Supplementary Table S1. List of indels in the seven *Ilex* species using *Ilex latifolia* as reference. [§]i means insertion, and d means deletion.

		**f**	**r**
Ilex	IRa-LSC	AGAGCCGGATCTAAGCGTTG	CTAGTAGAGGGGGCGGATGT
	LSC-IRb	TGGCCTACCATACGGTCTGT	GTCGGACAAGTGGGGAATGT
	IRb-SSC	AGGGAGAATACCGTCACCCA	CGGGAATAAGGGGATTGGCT
	SSC-IRa	TGTGGACAGGGACATCCTTC	ACTTCCGAAACGAAGGGGAC
Helwingia	IRa-LSC	AGAGCCGGATCTAAGCGTTG	CTAGTAGAGGGGGCGGATGT
	LSC-IRb	TGGCCTACCATACGGTCTGT	GTCGGACAAGTGGGGAATGT
	IRb-SSC	AGGGAGAATACCGTCACCCA	CGGGAATAAGGGGATTGGCT
	SSC-IRa	TGTGGACAGGGACATCCTTC	ACTTCCGAAACGAAGGGGAC

Supplementary Table S2. Primers validating four junction regions in resulting chloroplast genome of *Ilex pubescens* and *Helwingia himalaica*.

**Dataset**	**Length (bp)**	**Selected substitution models**
Complete chloroplast genome	161,798	TVM+G
Coding region	90,632	TVM+I+G
Introns and spacers	70,230	TVM+G
LSC	90,236	GTR+I+G
IR	26,161	TIM1+I+G
SSC	19,116	GTR+G

Supplementary Table S3. Length of each dataset matrix used for phylogeny construction and the best-fitting models tested by jModelTest for Bayesian Inference and Maximum Likelihood analyses.
